# Greater Cognitive Effort for Better Learning: Tailoring an Instructional Design for Learners with Different Levels of Knowledge and Motivation

**DOI:** 10.5334/pb.aw

**Published:** 2014-08-08

**Authors:** Seffetullah Kuldas, Lata Satyen, Hairul Nizam Ismail, Shahabuddin Hashim

**Affiliations:** 1 School of Educational Studies, Universiti Sains Malaysia, Malaysia; 2 School of Psychology, Deakin University, Australia

## Abstract

The capacity limitation of working memory is a widely recognised determinant of human learning. A cognitive load exceeding the capacity hampers learning. Cognitive load can be controlled by tailoring an instructional design to levels of learner prior knowledge. However, such as design does not necessarily motivate to use the available capacity for better learning. The present review examines literatures on the effects of instructional design, motivation, emotional state, and expertise level on cognitive load and cognitive effort, which ultimately affect working memory performance and learning. This examination suggests further studies on the effects of motivation and negative emotional states on the use of working memory. Prospective findings would help better explain and predict individual differences in the use of working memory for cognitive learning and task performance.

The capacity of human working memory is limited for conscious processing of novel information. Optimising the use of working memory is necessary for better learning, that is, for the construction of cognitive schemata — the classification of multiple elements of information into a single element and the storage of coherent knowledge structures in long-term memory (Sweller, Van Merriënboer, & Paas, 1998). Cognitive learning occurs better when learners mentally integrate novel information with prior knowledge, or include it into a relatively permanent storage of new coherent knowledge structures (Moreno & Mayer, 2007).

Empirical evidence indicates that imposing a high cognitive load (i.e., a large amount of information that exceeds or takes up valuable space of the capacity) is detrimental to the construction of cognitive schemata (Sweller, Ayres, & Kalyuga, 2011). To avoid such negative effects, excessive or redundant cognitive load needs to be controlled. Findings suggest that designing visual (i.e., figure, diagram, picture, or animation) and verbal (i.e., spoken or written text) instructional materials with respect to learners’ levels of prior domain-specific knowledge is a crucial measure to the control of cognitive load (Kalyuga, Ayres, Chandler, & Sweller, 2003). Such a measure would facilitate the prevention of an effective design (i.e., an integration or separation of verbal and visual instructional materials) from becoming ineffective; an instructional design effective for less experienced learners can be ineffective or harmful for more experienced learners, and vice versa (Kalyuga, 2008).

An instructional design however is not only aimed at controlling the cognitive load, but also at stimulating learners to use their available cognitive capacity for better learning (Paas, Tuovinen, Van Merriënboer, & Darabi, 2005). The amount of cognitive capacity that learners allocate to learning and task performance is referred to as cognitive effort (De Jong, 2010). Sweller (2010) suggests that encouraging learners to exert more cognitive effort is another measure crucial to the construction of cognitive schemata. Learners should be motivated to devote more cognitive effort to schema construction and automation to improve their cognitive task performance (Kalyuga, 2011; Paas et al., 2005; Schnotz & Kürschner, 2007). Without this motivation, an instructional design, which is solely aimed at controlling cognitive load, will remain insufficient to allow learners to exert necessary cognitive effort (Moreno, 2010; Paas et al., 2005; Schnotz, 2010; Van Merriënboer & Sweller, 2005). However, the extent to which motivational factors determine the use of working memory capacity needs to be clarified (De Jong, 2010). To shed light on this issue, Roets and Van Hiel (2011a) argued that the interplay between cognitive capacity, affect, and motivation should be taken into account.

This review paper primarily examines the use of working memory in relation with instructional design and learners’ levels of knowledge and motivation, so as to provide insights into two main issues: (a) how cognitive load and cognitive effort can be optimised via an instructional design, and (b) how cognitive capacity, affect, and motivation interact with one another and influence task performance. The examination of these issues serves to enhance the understanding of how learners are motivated to use their available working memory capacity to better construct cognitive schemata and perform cognitive tasks. The review concludes by suggesting the importance of conducting further studies on distinct motivational needs of learners, for the better use of working memory.

## Optimisation of Working Memory Performance through an Instructional Design

The term working memory refers to the human cognitive information-processing system that allows a combination of storage and manipulation of information (Baddeley, 2012). According to Baddeley, Allen, and Hitch (2010), working memory comprises four components. The central executive is the main component which acts as attentional control system for performing attention demanding cognitive tasks, allowing to focus attention on a task or to divide attention among concurrent tasks. It involves three temporary storage subsystems: (a) the visuospatial sketchpad to hold and manipulate spatial representations and visual images, such as shapes and colours, (b) the phonological loop to store and rehearse verbal and acoustic information, such as words or sounds, and (c) the episodic buffer to integrate visual and verbal information that is received from the other subsystems of working memory and from long-term memory. A substantial body of the literature provides evidence that working memory is limited in allowing the construction of new knowledge structures or their integration with prior ones in long-term memory. For example, Cowan (2001) demonstrated that working memory has a limited capacity, processing about four chunks of novel information at a time. Almost all the information is lost after about twenty seconds, if it is not intentionally rehearsed.

Due to the limitations on the storage and manipulation of novel information, simultaneously processing excessive verbal and pictorial information hampers learning (Sweller et al., 1998). To optimise or control cognitive load in working memory and facilitate learning, “Cognitive Load Theory (CLT)” has been widely applied to instructional design (see Sweller et al., 2011, for an overview of the recent version of CLT). According to CLT, the ultimate goal of an instructional design is to enable students to construct and automatise cognitive schemata. Van Merriënboer and Sweller (2005) remarked that “as is the case for schema construction, automation can free working memory capacity for other activities because an automated schema directly steers behaviour, without the need to be consciously processed in working memory” (p. 6). Working memory thereby handles complex materials which appear to exceed its capacity (Paas, Renkl, & Sweller, 2003). Thus, the more an instructional design could assist learners construct coherent cognitive schemata, the less they would be exposed to the limitations of working memory (Sweller et al., 2011).

CLT suggests that working memory is exposed to high cognitive load either due to the instructional design or the intrinsic complexity of a cognitive learning task; the former refers to “extraneous cognitive load” (ECL), and the latter refers to “intrinsic cognitive load” (ICL). Element interactivity (i.e., learning an information element, such as a concept or a procedure, with more or less reference to other elements) is the major source of both ICL and ECL (Pollock, Chandler, & Sweller, 2002). According to Beckmann (2010) and Sweller (2010), interactivity causing ECL can be reduced by altering instructional procedures, formats, or guidance; in contrast, interactivity causing ICL can be manipulated by altering the nature of the material that is learned.

As a result, manipulation of ICL and ECL is the central aim of CLT, which allows learners to optimise their cognitive performance by neither overloading nor underloading their working memory capacity (Sweller et al., 1998). However, an instructional intervention to control ICL or reduce ECL can hamper germane processes of learning (De Jong, 2010). To avoid such a detrimental effect, it should be clarified how and when the intervention contributes to schema construction and task performance.

## The Role of Learner Prior Knowledge

To predict when an instructional design hampers learning processes, Paas and Van Merriënboer (1994) argued that lower and higher levels of learner prior knowledge and task achievement should be considered. A substantial body of empirical evidence has substantiated this suggestion (e.g., DeLeeuw & Mayer, 2008; Kalyuga, 2007; Kalyuga & Renkl, 2007; Van Merriënboer & Sweller, 2005). According to Kalyuga (2008), “instructional techniques and procedures that are effective for novice learners may become ineffective, or even harmful, for more experienced learners, and vice versa” (p. 852). This change refers to “expertise reversal effect” (Kalyuga et al., 2003). In a series of studies (e.g., Kalyuga, Chandler, & Sweller, 1998; McNamara, Kintsch, Songer, & Kintsch, 1996; Yeung, Jin, & Sweller, 1997), readers who had insufficient knowledge about the content of a given text demonstrated a deeper comprehension of more elaborated text, while readers with sufficient knowledge showed a deeper comprehension of less elaborated text. Leung, Low, and Sweller (1997) similarly reported that supplementing a mathematical equation with extensive textual explanation did not improve learning for advanced students because the equation was intelligible in isolation. These findings suggest that an instructional intervention would be effective if it was separately tailored for low and high expertise students.

The desired effect of an instructional intervention on learners with different levels of prior knowledge can be achieved in an example-based learning environment (Reisslein, Atkinson, Seeling, & Reisslein, 2006). For instance, worked-example instruction is a promising technique that helps students develop problem-solving skills. “A worked-out example presents students with a problem statement, the worked-out solution steps that are necessary to solve the problem, and the final solution” (Moreno, Reisslein, & Ozogul, 2009, p. 83). In a series of studies (e.g., Kalyuga, Chandler, & Sweller, 2001; Kalyuga, Chandler, Tuovinen, & Sweller, 2001), students who learned from worked-out examples and thereafter performed their tasks were compared with those students who were allowed to explore the same task on their own. Less experienced students learned and performed better at a more difficult level, while there were minimal differences at the easier level. However, relatively more experienced students learned and performed better when exploring the tasks on their own rather than when practising the worked-out examples. In a further study (Reisslein et al., 2006), students with high prior knowledge learned better from problem-example pairs, because they studied the examples as feedbacks when they did not succeed in solving a problem within a certain time or certain number of attempts. By contrast, learners with low prior knowledge learned most from example-problem pairs. Thus, novice students’ learning may vary according to four conditions, namely example-only, example-problem pairs, problem-example pairs, and problem-solving-only. Van Gog, Kester, and Paas (2011) experimentally compared effects of these four conditions on novice students’ cognitive load and learning. Results of the prior knowledge test and the nine-point mental effort rating scale (developed by Paas, 1992) showed that novices learned most from the example-only and the example-problem pairs, thereby significantly outperformed those who practiced the problem-solving-only and problem-example pairs. Example-only and example-problem pairs were equally effective and efficient, whereas problem-example pairs did not lead to better learning than problem-solving-only. As a result, the abovementioned studies suggest that better learning can happen when novices or less knowledgeable learners are presented with example-problem pairs, but more knowledgeable leaners with problem-example pairs.

Effects of worked examples on learners with different levels of prior knowledge may also vary according to their types, namely product- and process-oriented examples. The product-oriented examples provide step-by-step solutions without explanations supporting each step, showing only the procedure for obtaining the final product. In contrast, the process-oriented examples contain statements that explain why each step is taken (Van Gog, Paas, & Van Merriënboer, 2004). Van Gog, Paas, and Van Merriënboer (2008) compared the relative effectiveness of both types of the example with learners at different levels of prior knowledge. They concluded that the process-oriented examples could be more efficient than the product-oriented examples, but only during the initial stages of learning. As the learning experience increased, the process-related information could become redundant, ineffective or even detrimental to learning (Van Gog et al., 2011). To avoid such inhibitory effects, an optimal transition from studying worked-out examples to solving problems independently is necessary (Renkl, Atkinson, Maier, & Staley, 2002; Van Merriënboer, Kirschner, & Kester, 2003). Contrary to less knowledgeable learners, those relatively more knowledgeable may benefit more from a rapid transition or immediately practicing problems after an introduction to a task (Sweller et al., 2011).

In order to facilitate a coherent construction of knowledge structures and an optimal transition from low to high expertise learners, Renkl and colleagues (Renkl & Atkinson 2003; Renkl, Atkinson, & Große, 2004; Renkl et al., 2002) proposed a “fading procedure” (successively fading of worked solution steps), gradually decreasing a higher level of problem-solving guidance and increasing problem-solving demands. The fading procedure describes a scaffolding of worked examples (Paas et al., 2003), providing learners with instructional supports that enable them perform a given task that they otherwise would not be able to perform successfully on their own (Vygotsky, 1978). Learners are initially required to study a fully worked-out example and, thereafter, complete partial worked-out examples, completing either the last (i.e., backward-fading) or the first (i.e., forward-fading) solution step of a problem practice, two solution steps of the second practice, three solution steps of the third practice, and so on, until they solve all steps (Moreno et al., 2009). The omitted steps are increased (i.e., removing the last or the first two, three, or more steps) until learners are able to complete all the steps of a problem solution on their own (Renkl & Atkinson, 2003).

According to Atkinson, Renkl, and Merrill (2003), backward-fading requires less time (imposing a lower cognitive load); therefore, it may be more efficient than forward-fading. However, on one hand, Moreno and colleagues (2009) showed that novice learners learned most from a forward-fading procedure and outperformed those studying a backward-fading procedure. On the other hand, Renkl and colleagues (2004) revealed that “it is not the position of the first faded step (forward or backward fading) that is crucial, but rather the type of the faded step that determines what is learned” (p. 66). They demonstrated that students learned problem-solving steps (i.e., basic principles of probability) that were faded (e.g., definition of probability, complementary rule, multiplication rule, addition rule) irrespective of the sequence of fading procedure. Renkl and colleagues (2004) concluded that “the backward procedure does not appear to offer any general advantage over the forward fading procedure” (p. 80). Further empirical studies are required for better insights into how to sequence the fading procedure and which step of solution should be faded first. Renkl and colleagues argued that “one must consider whether learning in a domain is best supported when knowledge about certain principles or solution steps is acquired first in order to facilitate further learning” (p. 80). As learners gain knowledge through a backward-fading procedure, further learning through a forward-fading procedure seems to be better (avoiding an extraneous cognitive load). In other words, novice or less knowledgeable learners may learn better through backward-fading, whereas relatively more knowledgeable learners may learn better through forward-fading.

Renkl (1999) argued that merely studying the worked-examples may not suffice to promote knowledge construction because novice learners may not be able to avoid misunderstanding the examples. They may also be unable to identify how the worked-out examples are relevant to corresponding learning tasks, such as problem-solving, or to use the same problem-solving steps to deal with new problems (Catrambone & Holyoak, 1989). Considering relatively more experienced learners, worked-examples may be perceived as dull and unchallenging as long as they experience no deficiency in their performance (Schnotz, Fries, & Horz, 2009). But when they first experience deficiencies during problem solving, they may be motivated to study related worked examples, revising the steps they could not solve (Reisslein et al., 2006). Novice learners, on their own, are less likely to demonstrate such effective use of worked examples as feedbacks and motivation to study the steps they could not solve; they need the necessary basic knowledge or guidance for accurately diagnosing their own performance deficiencies (Van Gog et al., 2011). To remedy an inhibitory effect of worked-out examples, learners need to be stimulated to give explanations about what steps are needed to solve a problem (i.e., self-explanation effect) and to establish a rationale for the problem-solving steps (Atkinson et al., 2003). However, self-explanation for complex tasks is suboptimal for novice learners (Kalyuga, 2007). Therefore, both low and high expertise learners should also deliberately engage in learning-practice activities, which is called the deliberate practice effect (Van Gog, Ericsson, Rikers, & Paas, 2005).

As Sweller and colleagues (1998) contended, an intervention effectively facilitates the construction and automation of schemata when it stimulates learners to engage in effortful learning, that is, to exert their available cognitive capacity for better learning and task performance as well as to repeatedly and successfully apply the acquired schemata to related learning tasks. However, effortful learning is not equally effective for high and low expertise students. Unlike experienced students, effortful learning may hinder rather than help less experienced students construct and automatise schemata (Schnotz & Kürschner, 2007). For instance, if low expertise students are asked to exert cognitive effort to imagine the content of worked-out examples before acquiring the related cognitive schemata, the imagination effect becomes an imposed high load, thereby inhibiting their learning (Cooper, Tindall-Ford, Chandler, & Sweller, 2001; Ginns, Chandler, & Sweller, 2003, Kalyuga, 2007; Leahy & Sweller, 2005). Hence, as these studies suggested, the demanded effort or difficulty level of a cognitive learning task and its instructional format should match the expertise level to facilitate schema construction and automation.

**Multimedia presentation.** Prior knowledge levels of learners appears to be central to learning from multimedia presentations. For instance, unlike students with sufficient prior knowledge, novice students need additional explanations for a multimedia presentation, such as onscreen text as supplement to an animation to explain a complex mathematical optimisation algorithm (Rey & Buchwald, 2011). Simultaneously presenting spoken and onscreen text for a diagram or positioning explanatory text and diagram spatially apart can cause novice students to split their attention, thereby inhibiting their learning processes (Ayres & Sweller, 2005). This “split-attention effect” impedes the mental integration and understanding of the relationship between the materials (Kalyuga, Chandler, & Sweller, 1999). To receive excessive information solely through visual sensory modality may overload the limited capacity of working memory, but simultaneous reception through both audio and visual modalities may expand the capacity. Kalyuga et al. (1999) highlighted that “dual-mode presentations do not reduce extraneous cognitive load, but rather increase effective working memory capacity” (p. 353). The audio-visual presentation, creating the modality effect, may particularly help low expertise students avoid a single channel overload or the split-attention effect (Kalyuga, 2012; Mayer & Moreno, 2003). As a result, learning processes of low expertise students can be hampered when they are provided with multimedia instruction regardless of the temporal or spatial contiguity between the visual and verbal materials (Mayer, 1999). For both low and high expertise students, the coherent construction of cognitive schemata is easier when pictorial presentations are accompanied with auditory explanations (spoken text) rather than onscreen text (Kühl, Scheiter, Gerjets, & Edelmann, 2011).

The advantage of audio-visual presentation over visual ones (onscreen text and diagrams or animations) for schema construction can be maintained, provided that the audio text is segmented into short lengths and accompanied by visually-presented instructional material, which are unintelligible without the text (Kalyuga, 2012; Leahy & Sweller, 2011). If the visual presentation is intelligible enough on its own, without narration or onscreen text, the simultaneous presentation of instructional materials may lead high expertise students to split their attention between the visual and verbal materials (e.g., diagrams and audio text) and overburden their working memory capacity. This is because visual and verbal working memory unnecessarily deals with the redundant text, thereby being exposed to “the redundancy effect” (Kalyuga et al., 1999). The onscreen text will be redundant if it merely reiterates the intelligible presentation and makes no significant contribution to the construction of cognitive schema. Kalyuga (2012) and Leahy, Chandler, and Sweller (2003) suggested that removing the redundant text can be beneficial to learning as students increase their expertise level, mainly because visual rather than textual presentation facilitates the mental integration process.

According to Moreno and Mayer (2007), schema construction and automation can be facilitated by considering several cognitive principles including: (a) presenting instructional materials that require the simultaneous operation of both audio-visual modality (i.e., modality principle); (b) respectively synchronising the audio-visual instructions in time and space (i.e., temporal and spatial contiguity principles); and (c) respectively excluding the material that is redundant or that does not contribute to instruction’s intelligibility (i.e., coherence and redundancy principles). However, such suggestions for dealing with the issue of how to optimise ICL and ECL by aligning an instructional design to learner expertise level fall short of providing a clear guidance on how to motivate learners of all expertise levels. Casting light on this issue requires further examination on how an instructional design would motivate learners to devote their available cognitive capacity for better learning and task performance (De Jong, 2010; Moreno, 2010; Schnotz & Kürschner, 2007).

## The Role of Learner Motivation

Research within the framework of cognitive load theory has aimed to allow learners to invest available cognitive effort in better learning. However, the influence of learner motivation has been neglected, despite its importance in deciding how much cognitive effort is invested (Ayres & Paas, 2012). More importantly, an investment of cognitive effort depends on perceived task difficulty, which can be a measure of both motivation and cognitive load (Schnotz et al., 2009). As a measure of higher or lower cognitive load, task-difficulty is often measured by an adapted version of the nine-point mental effort rating scale, developed by Paas (1992, see Van Gog and Paas, 2008, for a list of researchers who adapted this rating scale). As a measure of higher or lower motivation, the perceived probability of success along with other motivational subdimensions (i.e., anxiety, interest, and challenge) is often measured with the ‘Questionnaire on Current Motivation’, developed by Rheinberg, Vollmeyer, and Burns (2001). Both the questionnaire of actual motivation and the cognitive load scale includes “nearly the same kind of questions” (Schnotz, 2010, p. 318). Therefore, learners’ rates of both probability of success and cognitive load partially reflect the perceived task difficulty (Rey & Buchwald, 2011; Schnotz et al., 2009).

Using the abovementioned questionnaires, Rey and Buchwald (2011) tested if the expertise reversal effect could be explained by motivational factors (i.e., probability of success, interest, challenge, and anxiety), a cognitive load variable (i.e., redundancy effect), or both. The empirical result indicated a large correlation between “the probability of success” and “the redundancy effect”, indicating a partial overlap between these two variables. In contrast, there were only small to medium correlations between this cognitive load variable and the other three motivational subdimensions (i.e., interest, challenge, and anxiety). Rey and Buchwald (2011) concluded that differences in the redundancy effect between experts and novice learners, rather than differences in motivation (as single multidimensional construct), explain the expertise reversal effect. Generalizability of this finding is restricted to a specific cognitive load variable (i.e., redundancy effect), while the overlap suggests that motivational and cognitive explanations for the expertise reversal effect are not mutually exclusive in a classroom environment.

The allocation of cognitive effort to additional information processing is, however, not affected only by the probability of success or perceived task difficulty, but also by the perceived or expected costs of effort expenditure. Empirical evidence (Paas et al., 2005) suggests that if the effort expenditure is perceived as a waste or unnecessary for success, learners will not be motivated to exert sufficient effort. More cognitive effort will be invested when students perceive the effort expenditure to be necessary for better learning and task performance (Paas et al., 2005). Indeed, the evaluation process itself demands some time and effort, further drawing on motivational resources (Schnotz, 2010).

Moreover, when satisfactory success in a cognitive task is perceived to be challenging but probable and attainable, learners with low expertise would invest more cognitive effort. Less effort would be invested if the task is perceived to be either unchallenging or improbable and unattainable. In a relevant study by Schnotz and Rasch (2005), low expertise students demonstrated low performance in learning with animated pictures when they found a given task too easy. As the animation made the task unchallenging, they did not have to engage in more cognitive processing to construct mental representations on their own. Cooper et al. (2001) also confirmed that the presentation of animation usually lead low-expertise students to exert less cognitive effort and less time to construct mental representations and learn.

An unchallenging task can similarly reduce the persistence of high expertise learners in dealing with the task (Schnotz et al., 2009). Paas et al. (2005) showed that a multimedia presentation did not motivate high expertise learners to invest cognitive effort, because the task became unchallenging for them, whereas the opposite was true for relatively less-experienced learners. The multimedia presentation without onscreen text can be more challenging for expert, thereby leading them to invest more cognitive effort (Orvis, Horn, & Belanich, 2008). On the contrary, for low expertise learners, the animation without textual explanation can be complicated and frustrating, leading them to reduce their persistence (Schnotz et al., 2009). Therefore, if learners perceive a learning task as too easy or too difficult, they may not be motivated to invest their available cognitive effort for improved performance and learning (Paas et al., 2005; Schnotz, 2010; Schnotz et al., 2009).

To conclude, further clarifications are needed not only for the issue of how cognitive load is imposed and can be manipulated, but also of how to motivate students to devote their available cognitive capacity to coherent construction of cognitive schemata and better task performance. The necessary amount of motivational resources rather than the cognitive capacity to devote to performing a cognitive learning task can be an essential determinant of better learning (Moreno & Mayer, 2007). The relationship between the amount of cognitive load, the use of available capacity, and motivational factors in dealing with cognitive learning therefore needs further research (Moreno, 2010; Van Gog et al., 2005; Van Merriënboer & Sweller, 2005).

## The Interplay between Cognitive Capacity, Affect, and Motivation

According to Roets, Van Hiel, and Kruglanski (2013), the interaction between cognitive capacity and motivation depends on two conditions: (a) whether or not cognitive capacity is sufficient for deliberate processing of further information, and (b) whether or not a learner feels the need or has willingness to use the sufficient cognitive capacity to perform well on a cognitive task. Motivation for an investment of cognitive effort is determined by (a) perceived value of potential outcome of task performance, and (b) perceived probability of achievement of desired outcome if the investment for bolstering performance is increased (Brehm & Self, 1989). If the perceived value is relatively low, the motivation is likely to be fragile and disturbed easily by situational stressors, such as noise and time pressure. The stressors pose a burden on cognitive capacity, hamper performance and, thus, result in motivational decrements in investment (Roets & Van Hiel, 2011b). If the perceived probability is high, the potential effectiveness of increased investment is limited under prolonged exposure to aversive stressors (Roets & Van Hiel, 2011b).

According to Brehm and Self’s (1989) motivational intensity theory, individuals with high but not low motivation (willingness) opt to use their available cognitive capacity deliberately for processing further amount of information under stressors. This proposition prompts the question: does high motivation compensate for low cognitive capacity, and vice versa? Empirical evidence (Roets, Van Hiel, Cornelis, & Soetens, 2008) suggests that high but now low motivation may compensate for low cognitive capacity by leading to an increase in information sampling (additional information perceived to be useful for task performance). Roets and Van Hiel (2011b) found that when motivation is high for a task at hand, the investment of more cognitive effort can bolster task performance under stressors; but if motivation is low, stressors impair both performance and investment.

Roets and colleagues (2013) however maintained that high motivation can be inhibitory, rather than facilitatory, when available cognitive capacity is low. Pelham and Neter (1995) reported that high motivation is detrimental to performance in a cognitive task (e.g., mathematical problem-solving and recall tasks) when cognitive capacity is scarce, but improves the performance when cognitive capacity is sufficient. Roets and colleagues (2013) also demonstrated a similar finding when they compared low motivation (in a pilot study without requiring justifications for final judgment) with high motivation (in an actual study requiring justifications for final judgment) under two conditions: (a) low cognitive capacity, memorising a combination of three letters and three digits (e.g., AJY581), and (b) high cognitive capacity, memorising only one letter and one digit (e.g., E6). The joint impact of motivation and cognitive capacity (i.e., high motivation under high cognitive capacity) appeared to be beneficial for task performance but detrimental when motivation was high under low cognitive capacity.

The joint impact of motivation and cognitive capacity is also observable in two aspects of information processing modes: the peripheral versus the central processing mode or the heuristic versus the systematic processing mode (Roets et al., 2013). When both motivation and cognitive capacity are low, a student is likely to draw on the heuristic processing mode, thereby processing task-irrelevant information rather than task-relevant ones. Conversely, when both of them are high, a student is likely to draw on the systematic processing mode, processing task-relevant information rather than task-irrelevant information. Individuals can therefore maintain task performance (and compensate for reduced cognitive capacity) under stressors as long as they have high motivation and adequate cognitive capacity for further deliberate processing of information (Roets & Van Hiel, 2011b).

Roets and colleagues (2013) further maintained that the interaction effect between motivation and cognitive capacity on information processing is primarily determined by quality (i.e., subjective perception of usefulness of sampled information for task performance) rather than quantity (i.e., the amount of sampled information) of information. An increased amount of information as a result of motivation may negatively influence the perception of its usefulness (relevance, value, or informativeness) and thus be detrimental to task performance (Roets et al., 2013; Roets & Van Hiel, 2011a). However, as long as satisfactory performance in a cognitive task is perceived to be possible, high motivation leading to an increase in the amount of sampled information may, to some degree, compensate for low cognitive capacity (Roets et al., 2008, 2013). Finally, when a cognitive task becomes too difficult and success is no longer believed to be attainable or perceived as impossible, effort investment drops to minimal levels, regardless of motivation (see Roets et al., 2008).

## An Integrative Process Approach to the Interplay between Cognition and Motivation

Roets and Van Hiel (2011a) have proposed an “Integrative Process Approach (IPA)” to examine the interplay between the process variables, namely arousal, affect, motivation, cognitive ability, and motivation. IPA posits that a dynamic interplay (i.e., a causal link) between cognitive ability and motivation determines both the qualitative and quantitative value of information processing. A central aspect of IPA is that cognitive ability and motivation are “the most proximal process variables directly affecting information processing” (Roets & Van Hiel, 2011a, p. 510). According to IPA, higher levels of arousal (stress) and negative emotions reduce both information-processing time and attentional capacity, thereby altering the way information is processed, that is, the motivation for closure (Roets & Van Hiel, 2011a). The need for closure can be conceived as a desire to reach any answer that reduces or brings an end to aversive information processing (Kruglanski & Webster, 1996). Roets and Van Hiel (2008) have confirmed that individuals are highly motivated to reach closure when they perceive information processing as frustrating or aversive.

Roets and Van Hiel’s (2011a) review of evidence for affect as information suggested that affect itself triggers specific affect-related cognitions and defocuses attention, indicating an influence of affect on information processing through motivation. In other words, moods may evoke mode-congruent experiences stored in long-term memory; this retrieval takes up valuable space in working memory capacity (Roets & Van Hiel, 2011a). Depletion of available cognitive capacity may impede motivation, reducing willingness for processing further amount of information samples (Kruglanski & Webster, 1996). Roets and Van Hiel (2011b) demonstrated that in an initial phase of a decision task, negative affect reduced cognitive capacity, which, in turn, substantially decreased motivation for further information-processing in a latter phase of the task. Hence, “impaired performance may be the source rather than the result of investment decline” (Roets & Van Hiel, 2011b, p. 627). This finding suggests the causal influence of reduced cognitive capacity on motivation. The experience of inadequate cognitive capacity activates aversive feelings, which, in turn, may decrease motivation for task performance (Roets & Van Hiel, 2011b).

Overall in line with Roets and Van Hiel (2011a), the treatment of the process variables as if they operate in isolation is a too narrow approach to human behaviour (cognitive task performance). In particular, learners’ available cognitive capacity and willingness to use them are inseparable in a classroom setting. The dynamic interplay is likely to be the determinant of how learners allocate their cognitive resources to learning and task performance. For example, when a difficult task is perceived as burden by some novice students, it may lead to the need for closure. This assumption needs to be tested to explain and describe how instructional materials should be designed to avoid detrimental effects of the need for closure (i.e., avoiding a decrease in motivation for allocating more cognitive effort to task performance). Further empirical studies on impacts of the causal interaction between cognitive ability and motivation on effectiveness of an instructional design (i.e., on how learners use their cognitive capacity) should be conducted. An educational psychology research could apply IPA to instructional interventions (e.g., a design of instructional materials). Prospective findings would provide new insights into how to optimise the interaction between cognitive capacity and motivation (i.e., increasing motivation under sufficient cognitive capacity).

## Learner Motivation, Emotion, and Cognitive Effort

Researchers and educators who suggest innovative instructional interventions continue to face challenges in encouraging students to exert more cognitive effort for better learning and performance (Pintrich, 2003). Paas and Van Merriënboer (1994) contended that the difficulty level and instructional format of a cognitive learning task are determinants of cognitive load, whereas motivational factors are determinants of the devotion of cognitive capacity to better performance and learning. Paas and Van Merriënboer maintained that unless learners are motivated to invest cognitive effort, instructional manipulations do not effectively help with the utilisation of the available working memory capacity. Moreno and Mayer (2007) similarly stated: “when learners lack motivation they may fail to engage in generative processing even when cognitive capacity is available” (p. 315). Paas et al. (2005) showed that low motivation decreases cognitive effort and cognitive performance, while high motivation produces the opposite result. Hence, an instructional format needs to be motivating enough so that learners exert more cognitive effort for better performance.

Motivational factors, particularly those interwoven with emotional states, have been central to educational psychology studies (e.g., Harackiewicz, Barron, Tauer, & Elliot, 2002; Harackiewicz, Durik, Barron, Linnenbrink, & Tauer, 2008; Pintrich, 2003; Pintrich, Conley, & Kempler, 2003) when explaining how learners prefer to use their cognitive capacity, how some academically perform better compared to others, or how they adopt discrete achievement goals. According to the achievement goal theory developed by Dweck (1986) and Nicholls (1984) and related evidence (Elliot, 1999; Elliot & McGregor, 2001; Elliot & Murayama, 2008; Elliot & Thrash, 2001; Hulleman, Schrager, Bodmann, & Harackiewicz, 2010; Pintrich, 2000), four achievement goals – mastery-approach, mastery-avoidance, performance-approach, and performance-avoidance – energise and direct learner behaviour in task preparation and engagement. The reduced investment of cognitive effort is less likely to happen when approaching either the mastery or performance goal and more likely to happen with the performance-avoidance goals (Elliot & Moller, 2003, Pekrun, Elliot, & Maier, 2009; Senko et al., 2011).

Learners who approach the mastery goal with a high individual interest (considering learning activities and materials personally useful meaningful, valuable, or enjoyable) engage in more cognitive processing (Harackiewicz et al., 2008; Hidi & Renninger, 2006), but this engagement does not necessarily result in better academic performance (Harackiewicz et al., 2002). A series of studies (e.g., Graham & Golan, 1991; Levy, Kaplan, & Patrick, 2004; Linnenbrink, Ryan, & Pintrich, 1999) showed that learners pursuing the mastery goal devoted more cognitive effort to learn better. However, they similarly did not exert the effort to obtain a grade highly above the average class achievement, but only slightly above average. By contrast, learners approaching the performance goal drew on more cognitive effort to perform the task, attaining better class grades.

Mastery goal-oriented learners are not as compelled as those who are performance-focused to attend closely to the instructional material relevant to exams (Shell & Husman, 2008). Instead, they allocate more cognitive effort to studying individual learning interests (Senko & Miles, 2008). Senko and Miles (2008) reported that mastery-approach oriented learners prioritised their own individual interest to study and, therefore, paid little attention to instructional materials that were personally less interesting but required for exams. Conversely, performance goal-oriented learners devoted more cognitive effort to studying instructional materials that were very likely to be questioned on exams (Broekkamp & Van Hout-Wolters, 2007). Therefore, unlike mastery goal-oriented learners, those approaching the performance goal allocate less cognitive effort to studying individual learning interests, which are irrelevant to exams, and then generally achieve higher grades (Senko, Hulleman, & Harackiewicz, 2011).

Elliot (2006) noted that “goals are not sufficient to account for motivated behavior, it is also necessary to consider the motivation underlying goals” (p. 113) and that “a full account of motivation will attend to both direction (goal) and energization (the motivation underlying the goal)” (p. 114). Eccles and Wigfield (2002) suggested that some underlying motivational factors are associated with learner expectancy for success or failure and these are shaped by interlinked constructs of beliefs, such as self-efficacy, subjective value of the task, or perceived task difficulty, supporting the expectancy-value theory of achievement motivation. Evidence for these constructs generally indicates that individuals who believe they are able to learn or perform better exert more cognitive effort to achieve their subjective values compared to those who do not believe in their abilities and expect to fail or have low success (Bandura, Barbaranelli, Caprara, & Pastorelli, 2001; Eccles & Wigfield, 2002; Pintrich, 2003; Weiner, 2000; Wigfield & Eccles, 2000). However, consistently overestimating one’s own capability can also lead to disengagement from exerting cognitive effort to improve one’s weaknesses in a cognitive task (Bandura, 1997).

Apart from motivational factors that help improve learning and performance, a growing body of literature suggests that learners allocate some of their cognitive resources (i.e., duration and capacity of information-processing in working memory) to the expectancy for failure and task-irrelevant thoughts while performing a cognitive task (Kuldas, Hashim, Ismail, Samsudin, & Bakar, 2014). Task-irrelevant thoughts have been described as any attention-diverting thoughts that increase cognitive load and interfere with criterion task performance, including thoughts about one’s negative emotional state or other aspects not related to the task at hand (Seibert & Ellis 1991). According to the resource allocation theory (Ellis, 1990) and related evidence (Ellis, Moore, Varner, Ottaway, & Becker, 1997; Ellis, Ottaway, Varner, Becker, & Moore, 1997; Ellis, Varner, Becker, & Ottaway, 1995; Kliegel et al., 2005; Seibert & Ellis 1991), negative emotions (e.g., sadness and hopelessness) lead to an increase in task-irrelevant thoughts and diminish cognitive resources that are necessary for successful learning and task performance. Such effects can also occur when learners strive not to fall short of task mastery, avoiding skill decline, loss of existing knowledge, and learning failures (Conroy & Elliot, 2004; Van Yperen, Elliot, & Anseel, 2009); the effects also occur when learners strive not to do worse than others or appear incompetent (Grant & Dweck, 2003; Kaplan & Maehr, 2007). In these situations, anxiety levels increase, thereby leaving fewer cognitive resources to be allocated for better learning and task performance (Elliot & McGregor, 1999; Pekrun et al., 2006, 2009; Senko et al., 2011; Steele-Johnson, Beauregard, Hoover, & Schmidt, 2000; Tanaka, Takehara, & Yamauchi, 2006).

In a similar vein, the control-value theory of achievement emotions (Pekrun, 2006; Pekrun, Goetz, Titz, & Perry, 2002) suggests that individual differences in the devotion of cognitive effort to the achievement goals are associated with positive and negative emotions related to academic activities (i.e., task preparation and task engagement) and academic outcomes (i.e., lower or higher level of task performance). More cognitive effort for satisfactory learning and task performance is associated with positive emotions, such as enjoyment, pride, and hope, whereas the less cognitive effort is associated with negative emotions, such as boredom, anger, anxiety, hopelessness, and shame (Pekrun et al., 2006, 2009). Motives and emotions can therefore induce uneven devotion of cognitive effort to learning and performance of a cognitive task (Linnenbrink et al., 1999). Consequently, cognitive effort is not equally exerted for both the avoidance of expected failures and the attainment of success; the effort is usually decreased for the former and increased in the latter (Fisher & Ford, 1998).

As earlier discussed, another determinant of cognitive effort expenditure leading to low or high academic success is learner perception of difficulty or challenge in attaining an achievement goal (Grant & Dweck, 2003; Senko et al., 2011). The performance goal (normative goal) may be perceived as slightly challenging for some students, while extremely challenging for others (Blaga & Van Yperen, 2008); either of the cases usually leads to low investment of cognitive effort (Grant & Dweck, 2003). The more challenging and manageable a cognitive task is perceived to be, the more it increases pressure and induces greater effort to perform, enabling success on the task (Senko et al., 2011). Relatedly, Senko and Harackiewicz (2005) demonstrated that when both performance and mastery goal-oriented learners felt more pressure and perceived their goals as hard to attain or challenging (perceived goal difficulty), they focused their effort on task performance and performed better than those who pursued a standard mastery goal. The standard mastery goal was perceived as easier to achieve and therefore generated less pressure and produced poor task performance, although it aroused greater interest.

Accordingly, the challenging standard of the achievement goals is a motivational factor leading to individual differences in class achievements; the relatively poor performance of mastery-goal directed students has a relationship with the lack of challenge. Grant and Dweck (2003) found that academic performance proved better when the mastery goal-oriented individuals were challenge-seeking – not solely striving to improve learning. Thus, the perceived challenge to achieve a goal, but not merely the goal itself or individual interests can boost task performance. As Grant and Dweck concluded, “the impact of learning goals on performance may be seen chiefly when a high degree of challenge is present, when a task is personally important, or when the processing of complex, difficult material is necessary” (p. 550).

In conclusion, the relationship between achievement goals, task complexity, and instructional design merits attention to further explain whether cognitive load or the motivational goals best predict low task performance. Such a study might further provide a guideline for how the motivational goal and instructional design can be matched, so that learners can be encouraged to invest cognitive effort for better learning and task performance.

In addition, cognitive load as task-irrelevant thoughts need to be distinguished from the intrinsic and extraneous load and there is a need to clarify the extent to which learners have conscious control over the thoughts. Although they can consciously learn and motivate themselves to overcome failures in their learning processes, they cannot always be conscious learners, regardless of whether or not they are provided with an effective instructional design (Kuldas, Bakar, & Ismail, 2012). Learners do not have unlimited conscious control over the effects of their emotions, wishes, needs, and conflicting thoughts, or on their cognitive activities and behaviours while they evaluate, select, or classify information. Their cognitive responses to verbal and visual instructions are unlikely to be formed without mental representations influenced by unconscious emotional and motivational effects (Clark & Paivio, 1991; Kuldas, Ismail, Hashim, & Bakar, 2013). Therefore, to consider both conscious and unconscious learning processes would provide a deeper insight into the relationship between motivational factor, cognitive load, and cognitive effort (Kuldas et al., 2013). To focus only on cognitive limitations in working memory or in conscious learning deprive both educators and students of the unconscious contribution of motivational factors and limit the understanding of how human learning occurs (Kuldas et al., 2012).

## Effect of a Pleasant Learning Episode on Cognitive Effort

Learners’ cognitive effort is often based on their subjective perceptions or evaluations of both pleasant and unpleasant values of learning experiences (Kuldas et al., 2014). Such evaluations can vary according to affect experienced at the moment – instant utility, or in the past – remembered utility (Fredrickson, 2000). The remembered utility refers to “a global retrospective evaluation about the pain or pleasure associated with a past episode” (Hoogerheide & Paas, 2012, p. 887). Humans tend to evaluate or prefer their affective experiences with the greatest utility that maximise pleasure or minimise pain (Kahneman, Fredrickson, Schreiber, & Redelmeier, 1993).

Hoogerheide and Paas (2012) argued that the design of learning environments could be improved by taking the ‘remembered utility’ into account. Retrospective evaluation of a learning episode is mainly determined by how that episode felt emotionally when it was at its peak and when it ended. Hoogerheide and Paas measured (by a real-time measurement of experienced pleasure) the effect of the peak-end rule (i.e., pleasant learning episode, easier to learn and less tough to cope with) on the remembered utility and study behaviour. Primary school children were exposed to either (a) a list of 30 extremely difficult English–Dutch word pairs – high cognitive load (very unpleasant learning episode) – with a list of 21 moderate difficult word pairs (relatively more pleasant learning episode) at the end, or (b) a list of 30 easy-to-learn word pairs – low cognitive load (very pleasant learning episode) – with a list of 21 moderate difficult word pairs (relatively less pleasant learning episode) at the end. The learning episode with more pleasurable list of the word pairs at the end was evaluated as easier to learn and less tough to cope with; therefore, most participants preferred to restudy this episode. It can therefore be ascertained that the structure of learning tasks influenced the remembered utility. In this study, remembered pleasant (i.e., perceived low cognitive load) and unpleasant (i.e., perceived high cognitive load) learning experiences were determined by how learners felt when the learning experiences were at their peak and when ended. This finding, which is similar to Finn’s (2010), confirmed the so-called peak-end rule that remembered utility has an effect on both immediate and prospective study behaviour of students (i.e., their perception and preference of learning tasks – perceived high or low cognitive load).

The evidence for the peak-end rule (see also Finn, 2010; Fredrickson, 2000; Kahneman et al., 1993) appears to have implications for the measurement of cognitive load. The peak-end rule may influence learners’ experienced cognitive load during and after learning tasks (Hoogerheide & Paas, 2012). Taking into account such an influence may help researchers interpret ratings of cognitive load in the context of specific configurations of high and low cognitive load. Future research is needed to investigate how and when the remembered utility (the peak-end rule) in an instructional configuration of cognitive load facilitates cognitive and affective processes and outcomes of learning, such as task enjoyment, task engagement, task regulation, and task performance (Hoogerheide & Paas, 2012). Prospective findings would provide new insights into the relationship between the configuration of cognitive load and the investment of cognitive effort in terms of perceived pleasure of learning tasks and, thus, help develop a comprehensive measurement of cognitive load (i.e., precisely distinguishing between cognitive capacity and motivation in terms of perceived task-difficulty).

## Conclusion

To provide an insight into the use of working memory in relation to instructional design, expertise level, and motivation, this review has synthesised findings concerning two main issues: (a) how cognitive load and cognitive effort can be optimised through an instructional design, and (b) how cognitive capacity, affect, and motivation interplay with each other and thus impact on learning and task performance. The reviewed literature and cognitive load theory that are primarily concerned with the optimisation of cognitive load suggest tailoring an instructional design to learner expertise level. For example, learners with lower prior knowledge should be presented with example-problem pairs or with a spatial integration of visual and verbal instructional materials (e.g., auditory explanations for a diagram). This allows a manipulation of the intrinsic load. In contrast, learners having higher prior knowledge should be provided with problem-example pairs or with a spatial separation of the instructional materials (i.e., a reduction of the extraneous load). Such suggestions however fall short of explaining the optimization of invested cognitive effort. Although better cognitive learning requires an instructional design to optimise cognitive load, the allocation of more cognitive effort calls upon learner motivation. The reviewed evidence and the control-value theory of achievement emotions concerned with learner motivation and emotion show that the investment of more cognitive effort is associated with positive emotions (e.g., enjoyment and hope), whereas the investment of less cognitive effort is associated with negative emotions such as anxiety and hopelessness. The review has, therefore, concluded that an instructional intervention must be tailored not just to suit the expertise levels, but also to meet learners’ motivational needs for the optimisation of cognitive load and cognitive effort.

High motivation however can be inhibiting rather than facilitating task performance when cognitive capacity is insufficient. Both motivation and cognitive capacity should be sufficient or high in order to have the joint-facilitatory impact on learning and task performance. As such, it should be clarified how the desired impact can be created or how educators should design instructional materials in a manner to motivate learners to allocate more cognitive effort deliberately, particularly under situational stressors such as time pressure or noise. To clarify this issue, further in-depth exploration on the relationship between an instructional design and learner motivation is needed.

Most studies with the framework of cognitive load theory have thus far neglected the effect of learner affect and motivation on the use of cognitive capacity. Yet, cognitive load level is often determined by measuring perceived task-difficulty, which can be also the measure of learner motivation for the investment of cognitive effort. For instance, the perceived task-difficulty can be associated with the perceived pleasure of a learning task. Learners can perceive a learning task to be unpleasant (very difficult to learn, high cognitive load) at the beginning or during task performance, but pleasant (easier to learn, low cognitive load) at the end. In other words, the structure of learning tasks (starting to learn from more difficult to easier level of the task, or vice versa) can alter the perception of a learning episode, to be high or low cognitive load, according to how learners feel about the episode when it is at its peak and when it ends. Hence, the effect of perceived pleasure of a learning task (i.e., the peak-end rule) on the investment of cognitive effort needs to be considered.

As is proposed by the integrative process approach in this review, there is a dynamic interplay (a causal link) between cognitive ability, affect, and motivation that determines the qualitative and quantitative value of information (i.e., the perceived value of information and the processed amount of information). When information is perceived as frustrating or aversive, learners are highly motivated to reach closure (i.e., the need for closure), exerting no more cognitive effort for further processing of the information. Such affective values of information evoke affect-related cognitions and divert attention (i.e., reducing both information-processing time and attentional capacity). The resource allocation theory also confirms that negative emotions bring about task-irrelevant thoughts when moods or negative affect trigger mode-congruent experiences. Thus, retrieved task-irrelevant experiences from long term-memory leave fewer cognitive resources to be devoted to learning and task performance. This further diminishes learner willingness for further processing of the information. Such inhibitory effects of negative emotions can also occur when learners strive to avoid failures in learning and task performance. Hence, cognitive load as task-irrelevant thoughts associated with negative emotions need to be distinguished from the intrinsic and extraneous load.

Overall, this review suggests that cognitive effort, affect, and willingness of learners are not mutually exclusive in learning and task performance within a classroom environment. The dynamic interplay between learners’ cognitive ability, affect, and motivation determines their cognitive effort. However, further studies are required to cast light on the following issues: (a) how instructional materials should be designed to avoid detrimental effects (e.g., a decrease in motivation) of the need for closure, (b) how the causal interaction between cognitive capacity and motivation impacts effectiveness of an instructional design, (c) how and when the peak-end rule (remembered utility), in an instructional configuration of cognitive load, facilitates cognitive and affective processes and outcomes of learning, and (d) how available cognitive capacity and motivation interact in terms of perceived task-difficulty and perceived pleasure of a learning task. Further research could apply the integrative process approach to instructional interventions, in order to provide new insights into how the interaction between cognitive capacity and motivation could be optimised (i.e., increasing motivation under adequate cognitive capacity). Such research could also draw on theoretical frameworks concerned with the effect of emotions (e.g., the resource allocation theory) and achievement motivation (e.g., the achievement goal theory and the expectancy-value theory) on cognitive learning and task performance. Findings would also help explain, describe, and predict how learners devote their available cognitive capacity to task-relevant and task-irrelevant thoughts.
